# Knowledge, Attitudes and Practices Towards Diabetes Self‐Management Among People Living With Diabetes at Yumbe Regional Referral Hospital, Uganda

**DOI:** 10.1002/hsr2.73259

**Published:** 2026-09-14

**Authors:** Abeeho Tyson, Lwasa Faizo, Kabwagu Reagan, Banturaki Amon, Odong Patrick Olwedo

**Affiliations:** ^1^ Department of Internal Medicine Yumbe Regional Referral Hospital West Nile Uganda

**Keywords:** attitude, diabetes, knowledge, practice, self‐management, Uganda

## Abstract

**Background:**

Diabetes mellitus poses a growing public health challenge in Uganda and similar resource‐limited settings. Effective diabetes self‐management requires adequate knowledge, positive attitudes, access to care, medication adherence, dietary modification, physical activity, blood glucose monitoring, and foot care. Yumbe Regional Referral Hospital serves a diverse catchment population, including refugee communities, yet local data on diabetes‐related knowledge, attitudes, and self‐management practices remain limited.

**Objective:**

To assess diabetes‐related knowledge, attitudes, and self‐management practices among people living with diabetes at Yumbe Regional Referral Hospital and to identify selected sociodemographic and access‐related factors associated with self‐management practices.

**Methods:**

In a cross‐sectional study, 383 patients with diabetes were recruited from outpatient and inpatient settings. Data were collected through structured interviewer‐administered questionnaires and analyzed using SPSS v15, applying descriptive statistics, Pearson correlation, and multiple linear regression (*p* < 0.05).

**Results:**

Among 383 participants (mean age 53.9 years), most were Ugandan, male, married, and unemployed. Only 24.5% demonstrated good knowledge, while 41.8% had poor knowledge. Awareness of causes and complications was high, but understanding of types, hereditary risks, and suitable foods was limited. While medication adherence was strong (91.7%), only 30.5% monitored blood glucose once a week or more, 49.1% inspected their feet daily, and 59.7% engaged in physical activity at least three times per week. Cost and access were key barriers. Attitude was a stronger predictor of self‐care than knowledge.

**Conclusion:**

Patients at Yumbe Regional Referral Hospital demonstrated high medication adherence and generally positive attitudes, but important gaps remained in detailed diabetes knowledge, blood glucose monitoring, foot care, and access to support resources. Findings support structured, culturally appropriate diabetes education and improved access to self‐management supplies and follow‐up support. Because objective glycemic indicators were not collected, conclusions are limited to self‐reported self‐management practices rather than measured glycemic control.

## 1 Introduction

Diabetes mellitus (DM) is a chronic metabolic disorder characterized by persistent hyperglycemia owing to defects in insulin secretion, insulin action, or both [1]. DM is classified into four major categories, namely, type 1, type 2, gestational diabetes, and secondary diabetes [2]. The traditional paradigms of type 2 diabetes occurring only in adults and type 1 diabetes only in children are not accurate, as both diseases occur in all age‐groups [3]. In recent decades, the global prevalence of diabetes has risen dramatically, with estimates increasing from 108 million adults in 1980 to 537 million adults aged 20–79 years in 2021, with projections rising to 643 million by 2030 and 784 million by 2045 [4]. Diabetes is one of the leading causes of death worldwide and contributes substantially to cardiovascular diseases, kidney failure, and other disabling complications [5, 6]. The majority of the diabetes burden is now borne by low‐ and middle‐income countries, where resource constraints, limited health system capacity, and social determinants of health complicate its prevention and management [7].

Effective diabetes management extends beyond pharmacotherapy; it requires ongoing self‐care practices, medication adherence, dietary modification, regular physical activity, self‐monitoring of blood glucose (SMBG), and foot care paired with knowledge and positive attitudes towards disease control [8, 9]. Limited patient knowledge and negative attitudes are consistently associated with suboptimal self‐care behaviors and poor glycemic control [10, 11]. For instance, in several African settings, less than half of patients demonstrate adequate knowledge about diabetes and its self‐management, which contributes to delays in care seeking and higher rates of complications [12, 13].

In Uganda, facility‐based studies have documented widespread poor glycemic control. Patrick et al. [14] reported that approximately 84.3% of patients at the Mbarara Regional Referral Hospital had HbA1c levels ≥ 7%, indicating inadequate control and a high risk of complications. Population‐level surveys also showed a substantial burden of undiagnosed and poorly controlled diabetes [15]. Socioeconomic factors, health system weaknesses, and cultural beliefs influence knowledge and practices; low health literacy and misconceptions, such as viewing diabetes as a disease of the elderly only or relying solely on medications without lifestyle change, are frequently reported [16, 17].

Evidence from neighboring African countries underscores the variability of KAPs. For example, Joho et al. [13] reported that only 47% of patients in a Tanzanian multicentre sample had sufficient knowledge about diabetes, while Luambano et al. [12] documented a similarly low awareness of practical self‐care measures. Research from Ethiopia and other settings highlights the role of socioeconomic status, community support, and availability of diabetes education in shaping self‐care [18, 19]. Systematic reviews have emphasized that cost, limited access to supplies (glucometers and test strips), and fragmented health services are major barriers to effective self‐management in LMICs [7, 20].

Attitude and self‐efficacy are equally critical; patients who believe in the efficacy of lifestyle changes and who feel confident in their ability to self‐manage are more likely to adopt and sustain recommended behaviors [9, 21]. Conversely, low confidence and fatalistic beliefs correlate with poorer adherence and greater risk of complications [16, 22]. Behavior‐change frameworks, such as the Health Belief Model and Theory of Planned Behavior, have been used to explain these relationships and to design interventions that combine knowledge provision with motivational and social support components [21, 23].

Despite the increasing number of people living with diabetes in the West Nile sub‐region and at Yumbe Regional Referral Hospital, limited published data exist on patients' diabetes‐related knowledge, attitudes, and self‐management practices in this locality. Yumbe Regional Referral Hospital serves a catchment that includes refugees from Bidi‐Bidi settlement and populations across several districts, with contextual challenges including poverty, low literacy, cultural heterogeneity, transport barriers, and constrained access to specialized noncommunicable disease services. Local data are essential to inform context‐appropriate interventions that address specific knowledge gaps, attitudinal barriers, and structural constraints faced by patients in this setting. Therefore, this study assessed knowledge, attitudes, and self‐management practices related to diabetes management among patients attending Yumbe Regional Referral Hospital and examined selected sociodemographic and access‐related factors associated with self‐management practices. The findings are intended to inform tailored education, peer‐support, decentralization, and policy interventions for diabetes care in the West Nile region.

## 2 Methods

### 2.1 Study Design and Setting

This descriptive cross‐sectional quantitative study was conducted at Yumbe Regional Referral Hospital (YRRH), Yumbe District, West Nile, Uganda, from June 17, 2025 to August 19, 2025. Yumbe Regional Referral Hospital is a regional referral facility serving five districts and refugee populations from Bidi‐Bidi settlement. The hospital manages a growing number of patients with noncommunicable diseases, including diabetes, through outpatient NCD clinics and inpatient services.

### 2.2 Study Population and Sampling

The target population comprised individuals diagnosed with diabetes who attended the NCD clinic or were admitted to wards during the data collection period. A sample size of 384 was determined using Kish and Leslie's formula (1965) to estimate proportions with acceptable precision; 383 participants consented to participate and were enrolled. The original term “continuous random sampling” has been clarified as consecutive recruitment of eligible and consenting patients attending the outpatient NCD clinic or admitted on relevant wards during scheduled data collection days. To reduce selection bias, recruitment was conducted across different clinic days and ward visit periods during the study period. The inclusion criteria were patients with a diagnosis of diabetes who were clinically stable, available during data collection, and able to provide informed consent or assent with guardian consent where applicable. Patients who were too ill to participate, unable to communicate reliably, or experiencing severe diabetic distress were excluded.

### 2.3 Data Collection

Trained research assistants administered a structured interviewer‐administered questionnaire. The questionnaire was developed from the study objectives and informed by commonly assessed domains in diabetes KAP and self‐care tools, including knowledge of diabetes, attitudes toward self‐management, medication adherence, self‐monitoring of blood glucose, diet, physical activity, foot care, and perceived barriers. The instrument collected sociodemographic characteristics (age, sex, marital status, employment, monthly income, religion, residence, and distance to the nearest health facility), clinical history (age at diagnosis and care setting), knowledge domains (general concept, types, causes, heredity, management, complications, and recommended foods), attitudes (perceived importance, confidence, concern about complications, willingness to take medication and adopt lifestyle change, and perceived barriers), and self‐care practices (medication adherence, SMBG frequency, foot care, diet, physical activity, alcohol/tobacco use, access to support groups, and response to hypo/hyperglycemia).

The questionnaire was pretested before field data collection and administered in English or translated orally into Lugbara and Ma'di according to participant preference. Translation was performed by bilingual research assistants familiar with the local context. Formal psychometric validation, factor analysis, test‐retest reliability, and full forward‐backward translation were not conducted; this has been acknowledged as a measurement limitation.

### 2.4 Scoring

Knowledge scores were computed as the proportion of correct responses across all knowledge items and categorized using Bloom's cut‐offs as good (80%–100%), moderate (60%–79%), and poor (< 60%). Attitude items were summarized according to response categories reflecting perceived importance, confidence, willingness, concern, and barriers. Practice items were summarized by recommended self‐management behaviors, including regular medication use, blood glucose monitoring, correct response to hypo/hyperglycemia, physical activity, foot inspection, alcohol avoidance, and access to support resources. For interpretation, more frequent performance of recommended self‐care behaviors was treated as better practice. Because attitude and practice scales were not externally validated, the manuscript avoids overinterpreting these categories and recommends that future studies use validated tools or conduct full psychometric testing.

### 2.5 Data Management and Analysis

Data were analyzed using SPSS v15 for cleaning and analysis. Descriptive statistics (means, standard deviations, medians, frequencies, and percentages) summarized participant characteristics and KAP distributions. Bivariate analyses (*χ* ^2^ tests and *t*‐tests, where appropriate) explored associations between sociodemographic and access‐related variables and self‐management practices. Pearson's correlation coefficient assessed the relationship between knowledge and practice. A multiple linear regression model evaluated the influence of knowledge and attitude on practices, reporting *R* ^2^ and standardized beta coefficients; statistical significance was set at *p* < 0.05. Owing to limitations in available analyzable variables, the primary regression model included knowledge and attitude only. The omission of additional socioeconomic, access, and clinical covariates is acknowledged as a limitation because residual confounding and omitted‐variable bias may affect the estimated contribution of psychosocial factors.

### 2.6 Ethical Considerations

Ethical approval was obtained from the Gulu University Research Ethics Committee (GUREC) with approval number GUREC‐2025‐1211. Data collection was conducted from June 17, 2025 to August 19, 2025. Written informed consent was obtained from adult participants, and guardian consent was obtained for minors where applicable. Participation was voluntary, and participants were informed that refusal would not affect their care. Confidentiality was maintained by anonymizing questionnaires, avoiding personal identifiers in the analysis dataset, conducting interviews in as private a setting as feasible, and securely storing completed questionnaires. Research assistants were trained to use neutral wording to reduce social desirability bias, although interviewer‐administered data collection remains a limitation.

## 3 Results

### 3.1 Participant Characteristics

Of 384 eligible patients, 383 consented to participate (response rate 99.7%). The mean age was 53.9 years (SD 17.5), with ages ranging from 6 to 91 years and a median age of 56 years (Table 1). Minors constituted only 6 participants, representing 1.6% of the total sample; thus, their inclusion was too minimal to have any significant influence on the study results. Most participants were Ugandan nationals (88.3%), with males slightly outnumbering females at 55.1% and 44.9%, respectively. Most participants were married (74.7%), a small proportion were children (1.6%), and the majority were unemployed (61.9%); among those employed, self‐employment predominated (54.1%). Most identified as Muslim (65.0%) and resided in Yumbe district (72.8%). The nearest health facility for most respondents was a Health Centre III (32.1%). Monthly income was low: 44.6% reported earning less than 100,000 UGX, while only 3.7% reported earning > 500,000 UGX. The mean age at diagnosis was 45.6 years (range 7–88 years).

**Table 1:** Socio‐demographic profile of respondents (*N* = 383).

Variable	Category	Frequency	Percentage
Nationality	Ugandan	338	88.3%
	Non‐Ugandan	45	11.7%
Sex	Female	172	44.9%
	Male	211	55.1%
Marital status	Single	25	6.5%
	Married	286	74.7%
	Divorced/separated	24	6.3%
	Widowed	42	11.0%
	Not applicable	6	1.6%
Employment status	Employed	140	36.6%
	Unemployed	237	61.9%
	Not applicable	6	1.6%
Employment type (*n* = 157)	Self‐employed	85	54.1%
	Private sector	31	19.7%
	Government	26	16.6%
	Retired	15	9.6%
Religion	Muslim	249	65.0%
	Catholic	84	21.9%
	Anglican	37	9.7%
	Pentecostal	7	1.8%
	Seventh‐day Adventist	6	1.6%
District of residence	Yumbe	279	72.8%
	Moyo	28	7.3%
	Obongi	28	7.3%
	Adjumani	22	5.7%
	Koboko	23	6.0%
	Others	3	0.8%
Nearest health facility	Health centre III	123	32.1%
	Health centre IV	102	26.6%
	Health centre II	64	16.7%
	Regional referral hospital	61	15.9%
	General hospital	23	6.0%
	Clinic	10	2.6%

### 3.2 Knowledge Results

Using Bloom's categorization, 24.5% of respondents had good overall knowledge, 33.7% had moderate knowledge, and 41.8% had poor knowledge (Table 2).

**Table 2:** Overall classification of knowledge levels using Bloom's cutoff (*N* = 383).

Knowledge level	Bloom's cutoff score range	Frequency	Percentage
Very knowledgeable	80%–100%	94	24.5%
Moderately knowledge	60%–79%	129	33.7%
Not knowledgeable	< 60%	160	41.8%
Total		383	100.0%

Domain‐specific results showed near‐universal awareness of causes (99.7%) and high awareness of general management (99.5%) and complications (93.5%) (Table 3). However, knowledge regarding types of diabetes (59.3%), hereditary risk (45.4%), and foods appropriate for diabetes (63.4%) was notably limited. Alarmingly, 20.4% believed regular blood glucose monitoring was unnecessary.

**Table 3:** Domain‐specific knowledge about diabetes among respondents (*N* = 383).

Knowledge domain	Category	Frequency	Percentage
General understanding	Knowledgeable	339	88.5%
	Not knowledgeable	44	11.5%
Knowledge of causes	Knowledgeable	382	99.7%
	Not knowledgeable	1	0.3%
Knowledge of management	Knowledgeable	381	99.5%
	Not knowledgeable	2	0.5%
Knowledge of complications	Knowledgeable	357	93.5%
	Not knowledgeable	25	6.5%
Knowledge of types	Knowledgeable	227	59.3%
	Not knowledgeable	156	40.7%
Knowledge of good foods	Knowledgeable	243	63.4%
	Not knowledgeable	140	36.6%
Knowledge of hereditary nature	Knowledgeable	174	45.4%
	Not knowledgeable	209	54.6%
Belief in need for regular monitoring	Yes	305	79.6%
	No	78	20.4%

### 3.3 Attitudes

Most participants recognized the importance of diabetes management (78.9%) and expressed concern about complications (82.8%) (Table 4). Willingness to take prescribed medication was high (92.7%), and 86.6% reported readiness to adopt lifestyle changes. Nonetheless, 21.1% lacked confidence in their ability to manage diabetes and 39.1% disagreed that lifestyle modification alone could effectively manage diabetes. Cost of medications and supplies (44.4%) and limited access to healthcare services (36.8%) emerged as dominant perceived barriers.

**Table 4:** Attitudes towards diabetes management (*N* = 383).

Attitudinal statement	Response category	Frequency	Percentage
Attended diabetes education	No	177	46.2%
	Yes	206	53.8%
Importance of management	Very important	302	78.9%
	Somewhat important	63	16.4%
	Not very/not at all important	18	4.7%
Concern about complications	Concern to extremely concerned	317	82.8%
	Somewhat/not concerned	66	17.2%
Confidence in self‐management	Very/somewhat confident	302	78.9%
	Not very confident	81	21.1%
Has a management plan with doctor	No	206	53.8%
	Yes	177	46.2%
Willingness to take medications	Willing to extremely willing	355	92.7%
	Somewhat/not willing	28	7.3%
Willingness for lifestyle changes	Willing to extremely willing	332	86.6%
	Somewhat/not willing	51	13.4%
Belief in lifestyle management	Strongly agree	177	46.2%
	Somewhat disagree/strongly disagree	150	39.1%
	Neither	56	14.6%
Primary barrier faced	High cost	170	44.4%
	Limited access to healthcare	141	36.8%
	Lack of knowledge	41	10.7%
	Lack of motivation/support	24	6.3%
	No barrier	7	1.8%

### 3.4 Practices

Table 5 shows that medication adherence was high (91.7%). However, SMBG frequency was low: 69.5% monitored blood glucose once a month or less, while only 30.5% monitored once a week or more.

**Table 5:** Diabetes self‐management practices (*N* = 383).

Practice	Response category	Frequency	Percentage
Medication adherence	Always/most of the time	351	91.7%
	Sometimes/rarely/never	32	8.3%
Blood glucose monitoring	Once a month or less	266	69.5%
	Once a week or more	117	30.5%
	Never	0	0%
Action for high/low blood sugar	Correct	249	65.0%
	Incorrect	134	35.0%
Physical activity	≥ 3 Times a week	229	59.7%
	≤ 2 Times a week	154	40.3%
	Never	0	0%
Foot inspection	Daily	188	49.1%
	Less than daily/never	195	50.9%
	Never	0	0%
Alcohol consumption	Never	336	87.7%
	Ever (any frequency)	47	12.3%
Significant barrier	Cost	198	51.7%
	Lack of time	102	26.6%
	Access to healthcare	41	10.7%
	Lack of knowledge	34	8.9%
	Other	8	2.1%
Access to support resources	No	249	65.0%
	Yes	134	35.0%

Approximately 35% did not know the correct action in response to hypoglycemia or hyperglycemia. Foot care practices were suboptimal: 50.9% inspected their feet less than daily or never, while 49.1% performed daily foot inspections. Physical activity was reported by 59.7% of respondents at least three times per week, while 40.3% exercised two times per week or less. Alcohol consumption was low (87.7% never drink). Access to diabetes support groups was limited (35% had any access), and cost was the principal barrier to good practice (51.7%).

### 3.5 Associations and Predictors

Gender differences were observed in Tables 6 and 7, males more frequently reported proactive self‐care activities (frequent SMBG, regular physical activity, and foot inspections) and higher participation in support groups.

**Table 6:** Association between gender and practices.

Practices for diabetes management	Good practices	Gender		Total
		Male	Female	
I often take my diabetes medications as prescribed	Count	205	168	373
	% Within	55.0%	45.0%	
Days in the past week that you missed diabetes medications	Count	195	159	354
	% Within	55.1%	44.9%	
Medications or treatments currently used to manage diabetes	Count	211	172	383
	% Within	55.1%	44.9%	
I often check my blood glucose levels	Count	205	159	364
	% Within	56.3%	43.7%	
What I do when my blood sugar levels are either high or low	Count	130	119	249
	% Within	52.2%	47.8%	
I often engage in physical activity (e.g., walking, jogging) to manage my diabetes	Count	182	132	314
	% Within	58.0%	42.0%	
I often inspect my feet for signs of injury or infection	Count	145	113	258
	% Within	56.2%	43.8%	
I often consume alcohol	Count	182	154	336
	% Within	54.2%	45.8%	
Most significant barriers or challenges that prevent you from fully managing diabetes	Count	207	168	375
	% Within	55.2%	44.8%	
Access to diabetes support groups or resources you use to stay informed and motivated	Count	78	56	134
	% Within	58.2%	41.8%	
Total	Count	211	172	383

**Table 7:** Association between marital status and practices.

Self‐management practice	Marital status, *n* (%)				
	Single	Married	Divorced/separated	Widowed	Total
I often take my diabetes medications as prescribed	23 (6.3)	279 (76.0)	24 (6.5)	41 (11.2)	367
Days in the past week that you missed diabetes medications	21 (6.0)	268 (77.0)	23 (6.6)	36 (10.3)	348
Medications or treatments currently using	25 (6.6)	286 (75.9)	24 (6.4)	42 (11.1)	377
I often check my blood glucose levels	23 (6.4)	271 (75.7)	24 (6.7)	40 (11.2)	358
What I do when my blood sugar levels are high/low	13 (5.3)	187 (76.0)	13 (5.3)	33 (13.4)	246
I often engage in physical activity	21 (6.8)	232 (74.8)	21 (6.8)	36 (11.6)	310
I often inspect my feet for signs of injury or infection	18 (7.0)	188 (73.4)	21 (8.2)	29 (11.3)	256
I often consume alcohol	20 (6.0)	249 (75.2)	23 (6.9)	39 (11.8)	331
Most significant barriers or challenges	25 (6.8)	280 (75.9)	23 (6.2)	41 (11.1)	369
Access to diabetes support groups or resources	14 (10.6)	97 (73.5)	11 (8.3)	10 (7.6)	132
Total respondents in sample	25 (6.6)	286 (75.9)	24 (6.4)	42 (11.1)	377

Table 8 shows that income displayed a positive gradient with practices: higher income groups more frequently accessed support groups and reported better self‐care. Distance to healthcare facilities was inversely related to good practice: 63% of participants reporting good practices lived within 0–5 km of a facility, while very few living > 10 km away reported good practices (Table 9).

**Table 8:** Association between monthly income and practices.

Self‐management practice	Monthly income (in local currency), *n* (%)					
	< 100,000	100,001– 300,000	300,001– 500,000	> 500,000	Not applicable	Total
I often take my diabetes medications as prescribed	164 (44.0)	67 (18.0)	44 (11.8)	14 (3.8)	84 (22.5)	373
Days missed diabetes medications (past week)	153 (43.2)	66 (18.6)	42 (11.9)	13 (3.7)	80 (22.6)	354
Medications or treatments currently using	171 (44.6)	68 (17.8)	45 (11.7)	14 (3.7)	85 (22.2)	383
I often check my blood glucose levels	162 (44.5)	65 (17.9)	44 (12.1)	13 (3.6)	80 (22.0)	364
What I do when my blood sugar is high/low	100 (40.2)	46 (18.5)	32 (12.9)	7 (2.8)	64 (25.7)	249
I often engage in physical activity	131 (41.7)	59 (18.8)	39 (12.4)	13 (4.1)	72 (22.9)	314
I often inspect my feet for injury/infection	101 (39.1)	49 (19.0)	40 (15.5)	12 (4.7)	56 (21.7)	258
I often consume alcohol	149 (44.3)	54 (16.1)	43 (12.8)	12 (3.6)	78 (23.2)	336
Most significant barriers/challenges	166 (44.3)	68 (18.1)	44 (11.7)	13 (3.5)	84 (22.4)	375
Access to diabetes support groups/resources	43 (32.1)	37 (27.6)	27 (20.1)	5 (3.7)	22 (16.4)	134
Total respondents in sample	171 (44.6)	68 (17.8)	45 (11.7)	14 (3.7)	85 (22.2)	383

**Table 9:** Association between distance to health facility and practices.

Self‐management practice	Distance to nearest health facility, *n* (%)			
	0–5 km	6–10 km	> 10 km	Total
I often take my diabetes medications as prescribed	236 (63.3)	116 (31.1)	21 (5.6)	373
Days in the past week that you missed diabetes medications	224 (63.3)	112 (31.6)	18 (5.1)	354
Medications or treatments currently using	242 (63.2)	120 (31.3)	21 (5.5)	383
I often check my blood glucose levels	230 (63.2)	114 (31.3)	20 (5.5)	364
What I do when my blood sugar levels are high/low	143 (57.4)	88 (35.3)	18 (7.2)	249
I often engage in physical activity	196 (62.4)	98 (31.2)	20 (6.4)	314
I often inspect my feet for signs of injury or infection	165 (64.0)	78 (30.2)	15 (5.8)	258
I often consume alcohol	217 (64.6)	99 (29.5)	20 (6.0)	336
Most significant barriers or challenges	236 (62.9)	120 (32.0)	19 (5.1)	375
Access to diabetes support groups or resources	89 (66.4)	38 (28.4)	7 (5.2)	134
Total respondents (aggregate)	1978 (63.0)	983 (31.3)	179 (5.7)	3140

Pearson correlation showed a positive relationship between knowledge and practices (*r *= 0.294, *p*< 0.01) (Table 10). Multiple linear regression demonstrated that knowledge and attitude together explained 25.6% of variance in practices (*R* ^2 ^= 0.256, *p* < 0.001) (Table 11). Within the model, attitude had the larger standardized association (*β* = 0.420) compared with knowledge (*β* = 0.212). Because the model did not adjust for socioeconomic, access‐related, and clinical covariates, these findings should be interpreted cautiously.

**Table 10:** Correlation between knowledge and practices.

Variable	Pearson *r*	Spearman's rho	Significance
Knowledge versus practices	0.294**	0.254**	*p* < 0.01

*Note:* **correlation is significant at the 0.01 level.

**Table 11:** Regression analysis of knowledge and attitude on practices.

Predictor	*β* (Standardized coefficient)	*t*	Sig.
Knowledge	0.212	4.704	0.000
Attitude	0.420	9.310	0.000
*Model *R* ^2 ^= 0.256, *F*(2,380) = 65.486, *p *< 0.001*			

*Note: p* < 0.001 which indicates that the overall regression model is statistically significant.

Abbreviations: DM, diabetes mellitus; HbA1c, glycated hemoglobin; NCDs, non‐communicable diseases.

## 4 Discussion

This study assessed knowledge, attitudes, and self‐management practices among people living with diabetes at Yumbe Regional Referral Hospital in the West Nile region of Uganda. The revised framing focuses on self‐management practices because objective glycemic indicators such as HbA1c, fasting blood glucose, or random blood glucose were not collected or analyzed.

The study involved 383 respondents, predominantly Ugandan nationals (88.3%), with slightly more males (55.1%) than females (44.9%). Most participants were married (74.7%) and unemployed (61.9%), while over half (54.1%) of those employed (*n* = 157) were self‐employed. The majority were identified as Muslims (65.0%) and resided in the Yumbe District (72.8%), reflecting the cultural and demographic composition of the region. Health Centre III facilities were most commonly reported as the nearest health facility (32.1%), indicating reliance on primary healthcare services. The respondents' ages ranged from 6 to 91 years, with a mean age of 53.87 years (SD = 17.45) and a median age of 56 years, indicating a predominance of middle‐aged and older adults. Economically, nearly half (44.6%) earned less than 100,000 Uganda shillings per month, and only 3.7% earned more than 500,000 shillings, underscoring the low‐income levels within the study population. These findings are consistent with those reported by Ninsiima et al. [10] and Kasujja et al. [24] in Uganda, where the median age of the participants was 52 years (range: 45–60 years), with the majority (63.5%) aged between 36 and 59 years. Nearly half of the participants (47%) earned less than 100,000 UGX per month, and the vast majority (82.8%) lived within 5 km of the nearest health facility. These socio‐demographic findings highlight a community characterized by low income, high unemployment, strong religious and cultural identity, and reliance on primary health facilities. These characteristics have important implications for understanding health behaviors, service accessibility, and overall community well‐being in the study area.

The overall knowledge regarding diabetes management among respondents was generally low, with only 24.5% demonstrating good knowledge based on Bloom's cut‐off criteria. This level is lower than that reported in studies from other low‐income and middle‐income settings. For example, a study in Ethiopia by Mekonnen et al. [18] found that 37.8% of diabetic patients had good knowledge of diabetes self‐care, while Wolde et al. [19] reported 65.57% good knowledge of diabetes, suggesting that patients in Yumbe have comparatively poorer understanding, possibly reflecting differences in health education coverage, literacy levels, or access to diabetes care information in the region. Although respondents showed excellent awareness of causes (99.7%), management (99.5%), and complications (93.5%), their limited understanding of the types of diabetes (59.3%), dietary choices (63.4%), and hereditary nature (45.4%) mirror the patterns seen in other African contexts. For instance, Luambano et al. [12] observed that only 32.7% of respondents correctly identified types of diabetes, while in Turkey, Taskin Yilmaz et al. [25] found that only 62.7% knew that diabetes could be inherited. This indicates that while patients often grasp general concepts taught during diagnosis, specific knowledge that guides daily behavior, such as diet and heredity, remains poorly understood across various populations. A particularly concerning gap is the finding that 20.4% of respondents did not believe that regular blood sugar monitoring was necessary. Similar misconceptions have been reported in China, where Guo et al. [26] found that only 35.6% of participants monitored their blood glucose levels due to limited awareness or social support barriers. Such gaps highlight the urgent need for continuous patient education and follow‐up counseling to strengthen self‐management practices, especially in rural and resource‐limited settings.

The mean age at diagnosis (45.64 years) in this study aligns with findings from other settings where type 2 diabetes commonly presents in mid‐adulthood. Although participants demonstrated awareness of general diabetes concepts, gaps in diet, hereditary risk, types of diabetes, and blood glucose monitoring suggest that general awareness did not consistently translate into practical self‐management knowledge. These gaps may be related to limited structured diabetes education, low literacy, short counseling time during clinic visits, language differences, cultural beliefs, and reliance on informal health information. Community‐based education, health literacy initiatives, and regular diabetes counseling integrated into routine care may strengthen self‐management practices. Because measured glycemic outcomes were not available, this study cannot determine whether knowledge gaps translated into poor glycemic control.

While most participants had positive attitudes towards diabetes management, notable gaps remained in confidence, understanding, and access to care. The majority (78.9%) viewed management as very important, and 82.8% were highly concerned about complications, indicating a strong awareness of diabetes risks. More than half (53.8%) had attended diabetes education sessions, showing moderate exposure to health education. However, 21.1% lacked confidence in managing their condition, suggesting limited knowledge and inadequate support for self‐care. Although 92.7% were willing to take prescribed medications and 86.6% expressed readiness for lifestyle changes, nearly out of 10 (39.1%) disagreed that diabetes can be effectively managed through lifestyle adjustments. This reveals a significant misconception that may hinder the adoption of essential nonpharmacological measures, such as diet modification and regular exercise. Economic and systemic barriers also play a major role in shaping attitudes and behaviors. The high costs of medication and supplies (44.4%) and limited access to healthcare services (36.8%) were identified as the leading challenges, highlighting the structural inequalities that limit effective disease management. These factors likely contribute to frustration and decreased motivation among patients even when they recognize the importance of treatment. These findings are consistent with those of a number of recent studies. For example, a systematic review of lifestyle and dietary interventions in Africa identified both systemic (poor access to care, high cost, and limited health infrastructure) and personal barriers (low educational status, misconceptions, and poverty) as major obstacles to adherence [20]. In a South African study among type‐2 diabetic patients, high knowledge and positive attitudes towards lifestyle modifications were reported, but actual dietary practices remained poor, largely due to financial constraints and lack of adequate support at home [27]. Similarly, a qualitative study by Manyara et al. [22] found that cultural beliefs, limited education, low self‐efficacy, and economic hardship strongly impeded effective self‐management. The findings show that while people are eager to manage their diabetes, they face financial barriers, a lack of information, and systemic issues. To improve health outcomes, it is essential to emphasize patient education—particularly peer‐led programs and supportive social networks—and better training in self‐management and healthcare policies that make services more affordable and accessible. Tackling these aspects can turn positive intentions into effective diabetes management.

Self‐reported diabetes management practices among the 383 respondents revealed a high level of medication adherence (91.7%), but significant gaps existed in other critical areas. Notably, 69.5% monitored their blood glucose only once a month or less, 40.3% reported exercising two times per week or less, and 35% were unaware of the correct actions to take for high or low blood sugar.

Additionally, 50.9% performed foot inspections less than daily or never, and 65% lacked access to support resources. Cost was identified as the primary barrier to effective diabetes management by 51.7% of respondents. Comparatively, studies from Africa have highlighted similar challenges. In Ethiopia, a study found that only 42.4% of patients with type 2 diabetes adhered to recommended self‐care practices such as glucose monitoring and foot examination. However, the study also reported that factors such as membership in diabetes associations, possession of a home glucometer, and low perceived barriers were positively associated with adherence [21]. A systematic review of 12 studies in Ghana reported that adherence to self‐care behaviors among patients with type 2 diabetes remains suboptimal, with variations in adherence across different self‐care components. Foot care adherence ranged from 2.9 to 5.0 days per week, and self‐monitoring of blood glucose was performed on average 0.5–2.2 days per week [28]. The study revealed that, while patients generally adhered well to their medication, they struggled with blood glucose monitoring, some aspects of physical activity, and knowing how to respond appropriately to changes in their sugar levels. Diabetes care in Yumbe remains largely focused on clinical treatment rather than adopting a holistic approach. Shortcomings in foot care and insufficient social support highlight the importance of comprehensive diabetes management, which should include patient education, routine monitoring, community support groups, and programs supporting healthy lifestyles. Overcoming obstacles, such as cost, is essential for better self‐care and improved health outcomes.

The findings revealed clear socioeconomic and geographic disparities in diabetes self‐management practices. Respondents in the lowest income bracket (< 100,000 UGX) made up 40%–45% of those performing key self‐care behaviors, highlighting economic constraints that limit access to resources such as diabetes support groups, which increased from 32.1% in the lowest income group to 27.6% and 20.1% in the higher income brackets. Similarly, proximity to health facilities strongly influenced self‐management; approximately 63% of participants living within 0–5 km of a facility reported good adherence to practices such as medication intake, glucose monitoring, and foot inspection, compared to just 5%–6% of those residing more than 10 km away (Table 8). These findings are consistent with a Ugandan study by Ninsiima et al. [10] which also reported that low income and greater distance from health services were associated with poorer diabetes self‐care behaviors (Table 9). These findings highlight the significant impact of socioeconomic and geographic factors on diabetes self‐management in Yumbe. Individuals with lower incomes and those residing at greater distances from healthcare facilities demonstrated notably reduced adherence to essential management practices. These disparities indicate that poverty, elevated transportation costs, and restricted access to health information and support services are substantial barriers to effective diabetes care. Patients in remote locations tend to receive fewer educational opportunities and follow‐up sessions, while financial constraints may limit their ability to acquire necessary supplies such as glucometers and suitable footwear. Consequently, gaps in both knowledge and practice persist, especially among rural and economically disadvantaged groups. To address these challenges, this study recommends the implementation of community outreach programs aimed at delivering diabetes education closer to patients' residences, the integration of mobile health (mHealth) and peer‐support interventions, and the provision of subsidized or complementary diabetes supplies for low‐income populations. Additionally, strengthening primary healthcare systems and decentralizing diabetes services are advised to improve access to and continuity of care in this context.

The positive and statistically significant correlation between knowledge and diabetes self‐management practices in this study (Pearson *r* = 0.294, *p* < 0.01) aligns with the findings of Ninsiima et al. [11], who reported that good knowledge about glycemic control was associated with better self‐care behaviors among Ugandan patients with diabetes. This highlights the need to enhance patient knowledge, which is a crucial component for improving adherence to recommended practices and overall diabetes management.

The regression analysis indicated that knowledge and attitude were associated with diabetes self‐management practices, with attitude (*β* = 0.420) showing a stronger standardized association than knowledge (*β* = 0.212). This finding should be interpreted cautiously. The model did not include several potential confounders requested by reviewers, including education, income, distance to care, diabetes type, treatment modality, duration since diagnosis, and OPD/IPD status. Therefore, the apparent contribution of attitude and knowledge may be overestimated, while structural barriers may be underestimated. The findings nevertheless suggest that interventions should address both information gaps and psychosocial factors such as confidence, perceived barriers, motivation, and social support.

Furthermore, limited psychosocial support and cultural beliefs about diabetes may impact health attitudes. The study recommends implementing interventions that go beyond providing information and incorporating behavior change strategies such as motivational counseling, peer support, and community health education to encourage positive attitudes and empower patients. Including attitude‐based counseling in diabetes care along with regular follow‐ups and culturally appropriate educational materials may improve adherence and disease management in this context.

The strengths of this study include its relatively large sample, use of trained research assistants, and focus on an underrepresented referral‐hospital catchment population that includes refugee and host communities. Limitations include the cross‐sectional design, which precludes causal inference; reliance on self‐reported practices, which may be affected by recall and social desirability bias; interviewer‐administered questionnaires; lack of formal psychometric validation of the adapted questionnaire; absence of objective glycemic indicators such as HbA1c, fasting blood glucose, or random blood glucose; lack of stratified analysis by outpatient/inpatient status, diabetes type, and treatment modality; and a regression model that did not adjust for all socioeconomic, clinical, and access‐related confounders. The single‐center design also limits generalizability beyond the Yumbe Regional Referral Hospital catchment area.

## 5 Conclusion

The study concluded that people living with diabetes at Yumbe Regional Referral Hospital exhibited high medication adherence and generally positive attitudes toward diabetes management, but their overall knowledge and several self‐management practices remained suboptimal. Key gaps were identified in blood glucose monitoring, dietary knowledge, physical activity, foot care, and access to support resources, with low income and distance from health facilities emerging as important contextual barriers. Knowledge and attitude were associated with self‐management practices, although the absence of a fully adjusted multivariable model limits interpretation of their independent effects. These findings emphasize the need for targeted interventions that combine continuous patient education, psychosocial support, peer support, and improved healthcare accessibility to strengthen diabetes self‐management in this resource‐limited setting. Because objective glycemic measurements were not collected, the findings should not be interpreted as direct evidence of glycemic control.

### 5.1 Recommendations

Integrate structured, culturally sensitive diabetes education into routine care and community outreach, emphasizing practical self‐management such as diet, blood glucose monitoring, foot care, medication adherence, and response to hypo/hyperglycemia. Behavior‐change approaches such as motivational counseling, peer support, and family involvement should be incorporated to improve self‐efficacy. Diabetes services should be decentralized to primary health facilities and supported through community health workers to improve access for remote populations. Targeted subsidies or social protection may help low‐income patients access essential supplies, including glucometers, test strips, and appropriate footwear. Future studies should further investigate the root causes of poor diabetes self‐management in this setting, including socioeconomic, cultural, clinical, and health‐system barriers. Additionally, future longitudinal and interventional studies should assess the impact of these strategies on objective glycemic outcomes such as HbA1c and complication rates.

## Author Contributions

**Abeeho Tyson:** conceptualization, investigation, writing – original draft, formal analysis, methodology, data curation, writing – review and editing, visualization, validation, software. **Lwasa Faizo:** conceptualization, investigation, writing – original draft, visualization, formal analysis, writing – review and editing, methodology, validation. **Kabwagu Reagan:** conceptualization, investigation, writing – original draft, methodology, validation, visualization, formal analysis, writing – review and editing. **Banturaki Amon:** conceptualization, methodology, formal analysis, writing – review and editing, visualization, validation, supervision, data curation. **Odong Patrick Olwedo:** supervision, funding acquisition, project administration, resources.

## Funding

The authors have nothing to report.

## Conflicts of Interest

The authors declare no conflicts of interest.

## Transparency Statement

The lead/corresponding author Abeeho Tyson affirms that this manuscript is an honest, accurate, and transparent account of the study being reported; that no important aspects of the study have been omitted; and that any discrepancies from the study as planned (and, if relevant, registered) have been explained.

## Data Availability

The data that support the findings of this study are available from the corresponding author upon reasonable request, subject to applicable ethical and privacy restrictions.
